# The Indiana Association of Veterinary Graduates

**Published:** 1900-02

**Authors:** 


Secretary.

### THE INDIANA ASSOCIATION OF VETERINARY GRADUATES.

THE annual meeting was held at Indianapolis, January 3, 1900, with the following gentlemen in attendance: Drs. J. W. Klotz, T. A. Balser, O. L. Boor, J. E. Cloud, C. P. Wilson, G. H. Roberts, J. Crail, Geo. G. Ferling, Robert Harper, J. M. Patterson, A. W. Bitting, J. C. Rogers, L. A. Greiner, J. B. Heaton, I. D. Reynerson, F. W. Brewer, S. M. Springer, James Brashaw, J. M. Greiner, Dr. Mueller, and the visiting veterinarians from the Bureau of Animal Industry: Drs. N. C. Sorensen, Tait Butler, R. W. Tuck, and S. G. Hudson.

Dr. G. H. Roberts presented a paper upon "Parturient Paresis," giving his experience in a number of cases with the Schmidt treatment. The paper was fully discussed, and while the treatment has not been nearly so successful in the hands of those present as is frequently reported in the journals, it has been found superior to the old methods employed. Dr. Bitting reported in this connection that he had met a dairyman who for more than twenty-five years had treated his cases by washing out the udder with baking soda (bicarbonate of soda), a tablespoonful to the quart of water. His record of his cases showed that he had had good success as far as mortality was concerned, but had lost a large proportion of the udders with mammitis. He had used any kind of warm water, regardless of whether it had been boiled, and knew nothing of sterilizing the instrument, the injection fluid, or parts, which accounted for the unfavorable local effects.

Dr. L. A. Greiner read a paper upon "Fistulous Withers," and advocated bold operative procedures rather than the so-called conservative treatment.

Dr. T. M. Brewer discussed "Croupous Pneumonia."

Dr. J. O. Greeson sent two pathological specimens that were particularly interesting. One was a solid mucous cast of about three feet of the ileum and four inches of the cæcum, passed by a calf four months old. The case was catarrhal enteritis. The second specimen was a mucous cast of the prepuce of a steer affected with balanitis. Dr. Bitting renewed his plea for the preservation of choice pathological specimens and to make this exhibition a feature of the annual meetings. Through his efforts about three hundred specimens and photomicrographs of pathological tissues were made a part of the exhibit of comparative pathology at the State Medical Society last year and at the American Medical Association meeting. An attempt will be made to make the exhibit better this year.

This is the first meeting in five years in which the Indiana veterinarians have got down to work. Heretofore the question of legislation has received attention to the exclusion of other subjects, and with the effect of dividing the members. Indiana has only a little more than one hundred qualified veterinarians, distributed over seventy counties in the State, and the average distance which a man must go to obtain such service is about twelve miles.

Under such circumstances legislators will not consider a veterinary bill, and the Association has at last found it out.

Dr. Butler made a plea for a large attendance from this State at the forthcoming meeting of the American Veterinary Medical Association at Detroit. An effort will be made to get at least twenty to attend.

The officers for the coming year are: President, J. W. Klotz, Nobleville; Vice-President, Geo. G. Ferling, Richmond; Secretary, G. H. Roberts, Indianapolis; Treasurer, O. L. Boor, Muncie.

